# Understanding Human Cerebral Malaria through a Blood Transcriptomic Signature: Evidences for Erythrocyte Alteration, Immune/Inflammatory Dysregulation, and Brain Dysfunction

**DOI:** 10.1155/2020/3280689

**Published:** 2020-06-20

**Authors:** Sandrine Cabantous, Belco Poudiougou, Aurélie Bergon, Abdoulaye Barry, Aboubacar A. Oumar, Abdoulaye M. Traore, Christophe Chevillard, Ogobara Doumbo, Alain Dessein, Sandrine Marquet

**Affiliations:** ^1^Aix Marseille Univ, INSERM, UMR906, GIMP, Labex ParaFrap, 13005 Marseille, France; ^2^Aix Marseille Univ, INSERM, INRAE, C2VN, Marseille, France; ^3^Malaria Research and Training Center, Department of Epidemiology of Parasitic Disease, Faculty of Medicine, USTTB, BP 1805 Bamako, Mali; ^4^Aix Marseille Univ, INSERM, TAGC UMR U1090, 163 Av de Luminy, 13288 Marseille, CEDEX 9, France; ^5^Pediatric Wards, Gabriel Toure Hospital, Bamako, Mali; ^6^Centre des Oeuvres Universitaires, University of Bamako, BP 1805 Bamako, Mali; ^7^BILHI Genetics, Marseille, France; ^8^CNRS, Marseille, France

## Abstract

**Background:**

Cerebral malaria (CM), a reversible encephalopathy affecting young children, is a medical emergency requiring rapid clinical assessment and treatment. However, understanding of the genes/proteins and the biological pathways involved in the disease outcome is still limited.

**Methods:**

We have performed a whole transcriptomic analysis of blood samples from Malian children with CM or uncomplicated malaria (UM). Hierarchical clustering and pathway, network, and upstream regulator analyses were performed to explore differentially expressed genes (DEGs). We validated gene expression for 8 genes using real-time quantitative PCR (RT-qPCR). Plasma levels were measured for IP-10/CXCL10 and IL-18.

**Results:**

A blood RNA signature including 538 DEGs (∣FC | ≥2.0, adjusted *P* value ≤ 0.01) allowed to discriminate between CM and UM. Ingenuity Pathway Analysis (IPA) and Kyoto Encyclopedia of Genes and Genomes (KEGG) revealed novel genes and biological pathways related to immune/inflammatory responses, erythrocyte alteration, and neurodegenerative disorders. Gene expressions of CXCL10, IL12RB2, IL18BP, IL2RA, AXIN2, and NET were significantly lower in CM whereas ARG1 and SLC6A9 were higher in CM compared to UM. Plasma protein levels of IP-10/CXCL10 were significantly lower in CM than in UM while levels of IL-18 were higher. Interestingly, among children with CM, those who died from a complication of malaria tended to have higher concentrations of IP-10/CXCL10 and IFN-*γ* than those who recovered.

**Conclusions:**

This study identified some new factors and mechanisms that play crucial roles in CM and characterized their respective biological pathways as well as some upstream regulators.

## 1. Introduction

Malaria remains a major health problem worldwide with about half of the world's population at risk of infection, accounting for 3.2 billion people and resulting in approximately 219 million of cases annually (WHO, 2018). Although, most malaria cases are uncomplicated, a fraction of them (1%-3%), mostly young children, progress to severe disease, a life-threatening form which is characterized with higher mortality than UM. Approximately 435,000 people died of malaria in 2017, 91% of whom were residents of sub-Saharan Africa. Therefore, there is an urgent need for reliable diagnosis and efficacious treatment.

Cerebral malaria (CM) is one of the most severe forms of disease with a complex etiology. CM is a neurological complication due to *Plasmodium falciparum* infection that is caused in part by the sequestration of parasitized red blood cells in the microvessels of the brain, leukocyte adhesion to the microvasculature, cytotoxic lymphocyte activation, and an unregulated inflammatory response [1, 2]. Some key mediators of this inflammatory process including TNF-*α*, IL-6, IL-1*β*, IL-12, and IFN-*γ* have been involved in CM [3–6]. In addition, several polymorphisms have been associated with susceptibility/resistance to CM [7]. These polymorphisms are localized in genes having a role in three biological functions, the red blood cell physiology, the cytoadhesion of the infected red blood cells, and the immune response. But despite the advances and insights gained from genetic and immunological studies, our mechanistic understanding of physiological and molecular changes in human CM is still limited. Therefore, there is a need to make further progress in the understanding of pathogenic mechanisms and in the identification of molecular biomarkers to help for a better and faster medical care which would not only decrease the fatality rate but also reduce health-care costs and economic burden of the disease. As the molecular processes driving complex disease usually affect sets of genes acting in concert, one strategy is to use high throughput “omics” technologies to investigate in a pathological context several thousands of genes simultaneously. Hence, transcriptomic analysis is a powerful tool that can now be used to realize this previously unattainable goal in the study of complex infectious diseases. Recently, we carried out a study of the transcriptome of CM and UM children, from which we explored only some pathways and genes related to neurodegenerative diseases in order to validate our experimental approach [8]. Herein, we hypothesize that a whole gene expression profiling from peripheral blood may be robust enough to identify a specific molecular RNA signature of CM subjects allowing to elucidate the signaling pathways, gene interaction networks, and upstream regulators playing critical roles in the pathogenesis of CM. We also sought to identify new potential biomarkers for CM.

## 2. Materials and Methods

### 2.1. Ethics Review and Approval

This study was approved by the local ethics committees of the Faculty of Medicine, Pharmacy, and Odonto-Stomatology of the University of Bamako and by French ethics committees, including those of INSERM, the Comité de Protection des Personnes (protocol 212 CO2), the Ministère de l'Enseignement Supérieur et de la Recherche (protocol DC-2011-1426), and the Commission Nationale de l'Informatique et des Libertés (protocol 1564177). All experimental methods were performed in accordance with the Declaration of Helsinki. Written informed consent was obtained from all parents.

### 2.2. Patients

CM was defined according to the World Health Organization criteria as a state of unarousable coma with a Blantyre coma scale score of <2, a hematocrit of >16%, and parasitemia with asexual stages of *P. falciparum*. Meningitis was ruled out by lumbar puncture. Subjects with uncomplicated malaria (UM) had a thick blood film positive for *P. falciparum*, a Blantyre coma scale score of 5, and a hematocrit of >26%. The children with UM had never developed CM. Malian children were recruited through the Pediatrics Department of Gabriel Toure Hospital in Bamako, as part of a larger prospective field study. All blood samples were collected immediately after diagnosis on admission to hospital, and before treatment of the children.

### 2.3. RNA Extraction

A total of 13 patients with CM (male to female ratio, 7 : 6; mean age (±SD), 6.2 ± 3.8 years) and 12 patients with UM matched for age and sex (male to female ratio, 6 : 6; mean age (±SD), 6.5 ± 3.6 years) were selected for gene expression analysis; no patients died in these two groups. Among these samples, 7 CM and 8 UM were used for microarray studies. The samples were selected according to a very strict criteria and homogeneous phenotypes to obtain sufficient statistical power despite a limited number of samples. Peripheral blood mononuclear cells (PBMCs) were isolated within 1 hour after sample collection. Total RNA was extracted with TRIzol reagent (Life Technologies) according to the manufacturer's instructions but with an additional purification step performed with the RNA Clean and Concentrator kit (Zymo Research). High-quality RNA samples with integrity values of >8 (Agilent 2100 Bioanalyzer, Agilent technologies) were used as criteria to select subjects for microarray analysis (7 CM and 8 UM).

### 2.4. Whole-Transcriptome Analysis

The whole-transcriptome analysis was performed by using the Agilent technology, as previously described [8]. Each microarray contained about 22,700 genes based on RefSeq and 7,419 large intergenic noncoding RNAs. Fold-changes (FC) and *P* values were calculated for each gene, using the GeneSpring Software (Agilent). The moderated *t* test and the Benjamini-Hochberg correction for multiple tests were used to obtain the adjusted *P* values. Genes were considered significantly differentially expressed if presented an absolute FC between groups greater than 2.0 (∣FC | ≥2.0) and adjusted *P* value ≤ 0.05).

### 2.5. Bioinformatics and Statistical Analysis

Hierarchical clustering was carried out with the TMEV software 4.9.0 to show the distinguishable transcript expression patterns among samples using the average-linkage hierarchical clustering (HCL) with Pearson's correlation coefficient. The hierarchical clustering heatmap included the most significant probes (∣FC | ≥2.0) and adjusted *P* value ≤ 0.01 after the Benjamini-Hochberg correction. Canonical pathways, networks analysis, and upstream regulator analysis were performed with Ingenuity Pathway Analysis (IPA, Qiagen) [9]. Significant DEGs (∣FC | ≥2.0 and adjusted *P* value ≤ 0.05) were used for functional annotation by the IPA tool. This analysis allows us to determine the relevant biological pathways and other associations connecting the differentially expressed transcripts. Canonical pathways significant to the input data set were identified from the IPA library of canonical pathways based upon 2 parameters, (1) the ratio of the number of genes within the data set mapping to the pathway divided by the total number of genes mapping to the canonical pathway and (2) a *P* value (calculated based upon Fischer's exact test) determining the probability that each biofunction assigned to that dataset, and the canonical pathway is not due to chance alone. Canonical pathways are the idealized or generalized pathways that represent common properties of a particular signaling module or pathway. Networks were generated based on an algorithmically generated score based on connectivity between genes. A numerical value (*Z*-score) was used to rank networks according to how relevant they were to genes presented within the data set. Upstream regulators are the genes that affects the expression of numerous other genes. Network analysis reveals interactions between molecules in datasets presented as a graph. In addition, we also identified the significantly enriched KEGG pathways of DEGs (adjusted *P* value ≤ 0.05 and ∣FC | ≥2.0) using DAVID 6.8.

### 2.6. Quantitative Reverse Transcription Polymerase Chain Reaction (RT-qPCR)

We reverse-transcribed 400 ng of total RNA using the High-Capacity cDNA Archive kit (Applied Biosystems, Thermo Fisher Scientific) according to the manufacturer's protocol. The expression of selected genes was analyzed by TaqMan PCR, on an ABI 7900 real-time PCR thermocycler (Life Technologies), according to the manufacturer's instructions. The relative fold change obtained by qPCR of the candidate genes was determined using the 2^-*ΔΔ*Ct^ method after normalization with GAPDH.

### 2.7. Protein Quantification

The quantitative determination of IP-10/CXCL10 and IL-18 in the plasma of children was performed by ELISA (BD Biosciences and Abcys, respectively) according to the manufacturer's instructions. A total of 99 children was included corresponding to 17 fatal CM, 39 nonfatal CM, and 41 UM. The 17 children died several hours after their admission to the hospital. Unmatched groups were compared by the Mann-Whitney *U* test, with *P* value ≤ 0.05 considered to be significant. For the analysis of correlation between IP-10/CXCL10 and IFN-*γ*, the Spearman correlation coefficient test was used. Both tests were conducted by the use of SPSS (SPSS, version 10.1).

## 3. Results

### 3.1. Blood Transcriptomic Signature in CM

**Figure 1:**
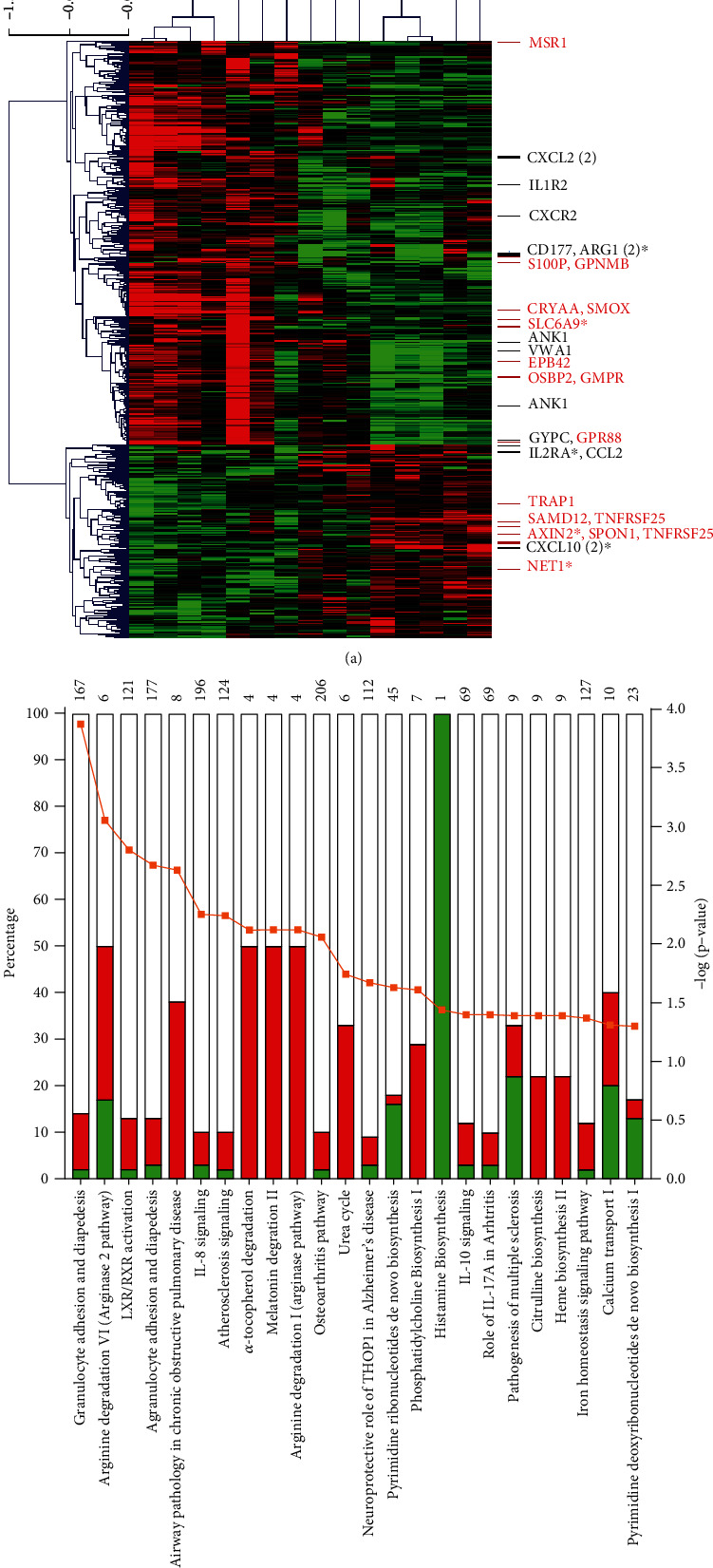
(a) Heatmap for differentially expressed genes between cerebral malaria (CM) and uncomplicated malaria (UM) children. Hierarchical clustering of microarrays was obtained using Pearson's correlation on probes with a fold change greater than two and adjusted *P* value ≤ 0.01. The red color represents high expression, while the green color represents low expression. The blue and purple bars at the top represent the CM and UM children, respectively. Some interesting candidate genes are indicated, in red for the new players of CM pathophysiology, in black for the genes previously described in malaria. (b) Stacked bar chart displaying the top canonical pathways found to be differentially represented in comparing gene expression in CM and UM children absolute fold change ≥2, adjusted *P* value ≤ 0.05. The total number of genes in each pathway is displayed above each bar. Pathways are ranked by statistical significance; the orange dot indicate the -log (*P* value). The percentage of dysregulated genes is indicated for each pathway, downregulated genes are in green, and the upregulated genes are in red. The ratio of the numbers of DEGs to the total number of genes in the pathways ranged from 9% to 100%.

A total of 7 CM and 8 UM subjects with very good RNA quality were used to perform gene expression profiling analysis. This analysis revealed that there were 1,366 significantly differentially expressed probes between CM and UM with an absolute fold change ∣FC | ≥2.0 and adjusted *P* value ≤ 0.05 after the Benjamini-Hochberg correction. Accordingly, we identified 1,044 upregulated and 322 downregulated probes between CM and UM. A hierarchical clustering heatmap (Figure 1(a)) demonstrated the specific expression of the 538 most significant probes (∣FC | ≥2.0 and adjusted *P* value ≤ 0.01 after the Benjamini-Hochberg correction) and revealed the degree of separation between CM and UM subjects. Of these probes, 432 were upregulated and 106 were downregulated. This hierarchical clustering allowed us to get an RNA signature that discriminates between CM and UM (Figure 1(a)). Interestingly, among the differentially expressed genes (DEGs) that make up this signature, some had never been previously involved in malaria pathogenesis and/or severity. Notably, the upregulated genes GPR88, EPB42, GPNMB, S100P, GMPR, CRYAA, OSBP2, SLC6A9, MSR1, and SMOX as well as the downregulated genes SAMD12, SPON1, IL12RB2, NET1, AXIN2, IL18BP, TNFRSF25, and TRAP1 have attracted our attention because of their biological function (Figure 1(a), Table 1). Of note that among the genes included in this signature, some have already been involved in the development of clinical forms of malaria, and either encode for proteins of erythrocytic surface membrane ANK1 and GYPC (Table 1) or for inflammatory/immune components CXCL10, CCL2, CXCR2, VWA1, CD177, IL1R2, ARG1, IL2RA, and CXCL2 (Figure 1(a), Table 1).

### 3.2. Canonical Pathway Enrichment Analysis of DEGs

In order to gain a greater insight into specific pathophysiological process, enriched pathway and functional classification analyses of DEGs were performed using IPA. This analysis was conducted with the DEGs exhibited ∣FC | ≥2 and adjusted *P* value ≤ 0.05 after the Benjamini-Hochberg correction. A total of 24 canonical pathways were significantly altered (Figure 1(b)). Interestingly, the top 4 canonical pathways were the granulocyte adhesion and diapedesis with 12% (20/167) and 2% (4/167) genes upregulated and downregulated, respectively, in CM, the arginine degradation (33% up and 17% down), the LXR/RXR activation (11% up, 2% down), and the agranulocyte adhesion and diapedesis (10% up and 3% down). Additional significant pathways were also of particular interest such as IL-8 signaling (6^th^ ranked pathways in terms of significant), the neuroprotective role of THOP1 in Alzheimer's disease (13^th^), IL-10 signaling (17^th^), and role of IL-17A in Arthritis (18^th^) because of their involvement in brain disorders and inflammatory/immune response. Of the significant genes that overlap with the above-listed canonical pathways, the great majority (146 versus 51) showed higher expression in the patients who developed CM as compared to those who had UM. Among these genes, some were previously shown to play a role in severe malaria, specially CXCL10, ARG1, ATP2B4, and MMP9. MMP9, ARG1, and CXCL10 were common to at least 3 of the 24 significant canonical pathways. Of these, MMP9 and ARG1 were upregulated in CM while surprisingly CXCL10 was downregulated.

### 3.3. KEGG Pathway Enrichment Analysis of DEGs

A KEGG pathway enrichment analysis based on the 1,366 significant probes with ∣FC | ≥2 (of these 994 with genes ID) revealed that the “malaria” pathway (hsa05144) was the first most significant. Among the top 7 pathways, six have been previously identified by others using transcriptome analysis in malaria [10, 11], including malaria (hsa05144), cytokine-cytokine receptor interaction (hsa04060), TNF signaling pathways (hsa04668), chemokine signaling pathways (hsa04062), *Salmonella* infection (hsa05132), and cell cycle (hsa04110). Importantly, the novel identified pathway was a “NOD-like receptor signaling pathway” (has04621) which highlights the key role of the inflammasome in the CM development.

### 3.4. Interaction Network Analysis

**Figure 2:**
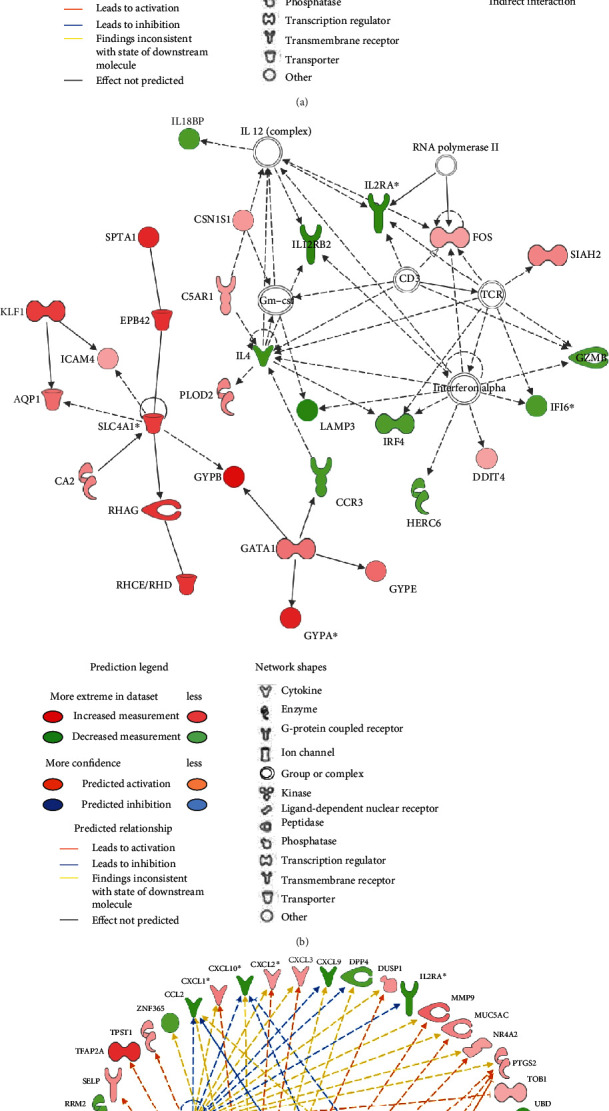
Gene network and upstream analysis by Ingenuity Pathway Analysis (IPA; Qiagen Inc.). Gene network highlighting the candidate genes and their interaction with the genes presented as nodes and relationship between two indicated as a line. Upstream regulator analysis allows the identification of transcriptional regulators and their target genes dysregulated in our dataset. (a) Gene network for connective tissue development and function, tissue morphology, and connective tissue disorders. (b) Gene network for cell death and survival, connective tissue disorders, and hematological disease. (c) The transcriptional regulator TNF was ranked first among the upstream regulators, and 38 genes were predicted to be activated or inhibited by TNF. Four additional upstream regulators SLC4A1, IL-1B, STAT3, and IL-6 have been identified.

**Figure 3:**
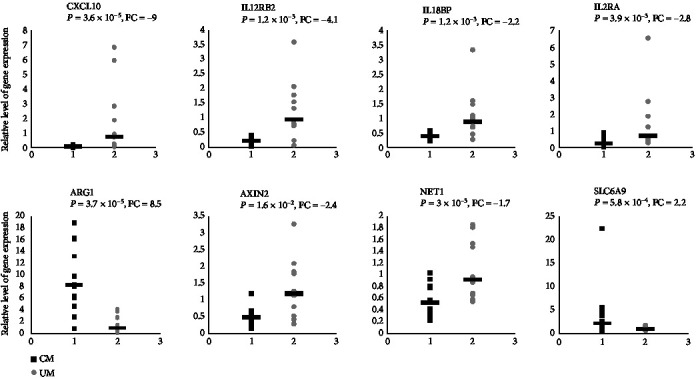
Real-time polymerase chain reaction-based validation of messenger RNA levels for 8 significantly dysregulated genes between CM and UM. Samples from 13 children with cerebral malaria (CM) and 12 with uncomplicated malaria (UM) were analyzed. Relative expression levels were calculated from 2^-*ΔΔ*Ct^ values. Values for children with CM are represented by black squares, and values for children with UM are represented by gray circles. The horizontal lines indicate median values. We used the Mann-Whitney *U* test to compare the results for the CM and UM groups.

To discover potential novel regulatory networks and causal relationship associated with our DEGs, we have built and explored transcriptional networks using IPA. We obtained 5 interactive gene networks to be significantly associated with CM (IPA score > 20): (1) connective tissue and development and function, tissue morphology, and connective tissue disorders (score = 36) (Figure 2(a)), (2) cell death and survival, connective tissue disorders, and hematological disease (score = 36) (Figure 2(b)), (3) digestive system development and function, organismal development (score = 30), (4) cellular movement, inflammatory response, and cell-to-cell signaling and interactions (score = 23), and (5) immunological and dermatological disease and conditions and inflammatory disease (score = 23). The top 2 network diagrams indicated that most of DEGs are apparently involved in the host response and erythrocyte development and function. Among genes of network 1, only one were found to be downregulated (CCL2) and 28 genes were upregulated (Figure 2(a)). Collectively, the group of upregulated genes of the network 2 is predominated by genes encoding for the red blood cell proteins with SLC4A1 as hub gene whereas the group of downregulated genes with IL-4 as hub gene was involved in the immune response (Figure 2(b)). Interestingly, IL-18BP (interleukin 18-binding protein), known to encode a decoy receptor for IL-18, was downregulated. Importantly, this cytokine whose expression is mediated by inflammasome has important functions in innate and adaptive immunity.

### 3.5. Upstream Regulators

Based on IPA, five upstream regulators including TNF (*P* = 1.4 × 10^−7^), IL-6 (*P* = 2.3 × 10^−4^), IL-1B (*P* = 1.4 × 10^−5^), STAT3 (*P* = 5.3 × 10^−5^), and SLC4A1 (*P* = 8.6 × 10^−6^) were involved in regulating DEGs in CM (Figure 2(c)). TNF, IL-6, IL-1B, and STAT3 were not dysregulated in our dataset whereas SLC4A1 was upregulated in CM. A total of 55 genes were affected by at least one upstream regulator; of these, 39 and 16 were upregulated and downregulated, respectively.

### 3.6. Microarray Data Validation by RT-qPCR

A total of eight candidate genes were selected from the RNA signature for qPCR validation. We have deliberately chosen genes with an increase (ARG1 and SLC6A9) or a decrease (IP-10/CXCL10, IL-12RB2, IL-18BP, IL-2RA, AXIN2, and NET1) in gene expression in CM children (Table 1 and Figure 2(b)) by including genes recently shown to be involved in CM. Most of the selected genes are involved in the inflammatory response which plays a critical role in CM as shown here. All the selected genes showed significant changes between CM and UM consistent with those observed by microarrays (Figure 3).

### 3.7. Plasma Concentration of IP-10/CXCL10 and IL-18

**Figure 4:**
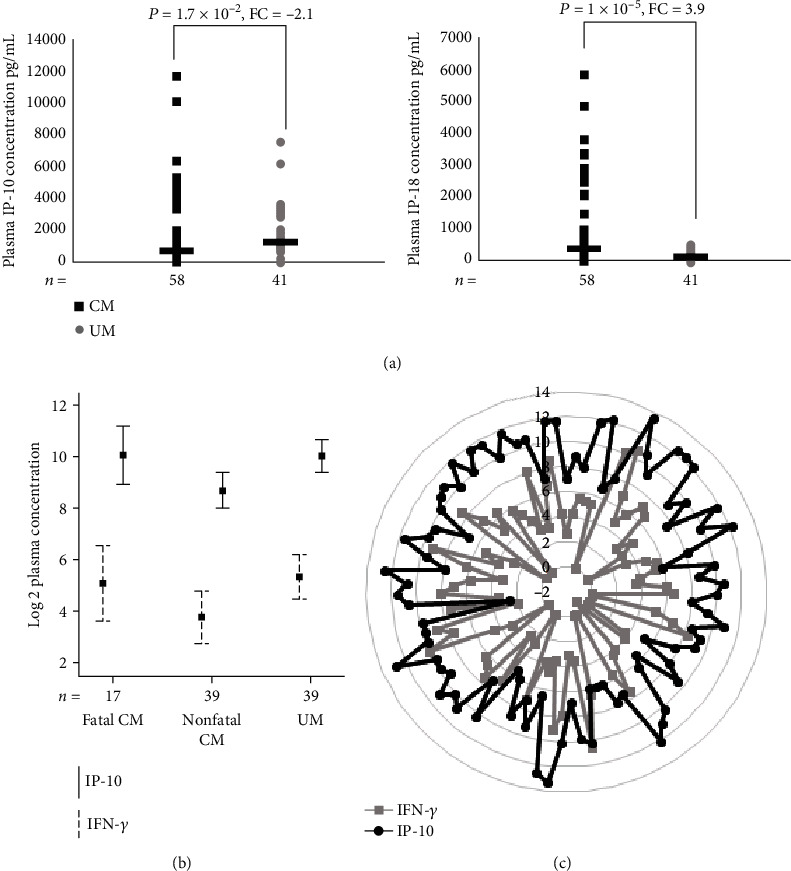
(a) Plasma IP-10 and IL-18 concentrations in children with CM (*n* = 58, black squares) and UM (*n* = 41, gray circles). The nonparametric Mann-Whitney *U* test was used to assess differences. (b) Plasma IP-10 and IFN-*γ* concentrations in children with fatal CM and nonfatal CM and UM. The levels are represented in log. (c) Plasma IP-10 and IFN-*γ* concentrations for each child with CM or UM. The levels are represented in log. We used Spearman's correlation coefficient.

Surprisingly, we observed a lower transcription level of IP-10/CXCL10 in CM compared to UM at the admission to the hospital both by microarray and RT-qPCR whereas most studies suggested CXCL10 as risk factor in severe disease. To determine whether the protein level was consistent with the transcript level, we measured plasma IP-10/CXCL10 concentration (Figure 4(a)). In CM, the median plasma IP-10/CXCL10 concentration was significantly lower than that in UM (583 and 1,250 pg/mL, respectively; *P* = 1.7 × 10^−2^). Among the children with CM, those who died of the complication of malaria tended to have higher IP-10 concentration than those who recovered (962 versus 440 pg/mL; *P* = 5.5 × 10^−2^) (Figure 4(b)). The median IP-10 concentration was 1,250 pg/mL in the children with UM and was 440 pg/mL (*P* = 2 × 10^−3^) in those who recovered from CM. Because IP-10 is a chemokine secreted from a variety of cells in response to interferon-*γ* (IFN-*γ*), we tested the correlation between the plasma levels of IP-10 and IFN-*γ* in a total of 95 children (Figures 4(b) and 4(c)). The analysis revealed a significant positive correlation between IP-10 and IFN-*γ* plasma levels when it was performed on overall children (Spearman's correlation coefficient, *r* = 0.475; *P* = 1 × 10^−6^). A positive significant correlation between both protein levels was also observed among fatal CM (*r* = 0.584; *P* = 1.4 × 10^−2^), CM who recovered (*r* = 0.492; *P* = 1 × 10^−3^), and UM (*r* = 0.402; *P* = 1.1 × 10^−2^). We also quantified plasma IL-18, a cytokine known to be an inducer of IFN-*γ* and Th1 response [12]. IL-18 concentrations were significantly higher in children with CM (median, 338 pg/mL) than in UM (median, 86 pg/mL) (*P* = 1 × 10^−5^) (Figure 4(a)). Interestingly, whereas the plasma concentration of IL-18 was higher in children with CM, the gene expression of IL-18BP was lower in these same children.

## 4. Discussion

Because the interactions between biomolecules play crucial roles in disease development, the understanding of the topology of biological networks and pathways is likely to be the most powerful strategy to find molecules that may have biological and clinical applications. However, deciphering the complex regulatory processes of pathophysiological pathways in human brain remains a challenge due to the inaccessibility of antemortem tissue. Fortunately, the studies on peripheral blood can provide important mechanistic knowledge that may have therapeutic implications [13–15] as shown previously into various infectious and neurological diseases [16–19]. Hence, we speculated that multiple dysregulated pathways may favor CM and that we are able to identify most of them in peripheral blood. We performed gene expression profiling using microarrays of whole-blood samples from CM and UM children, and we showed that our data were robust enough to detect new players of CM.

We first evaluated the transcriptional profiles by hierarchical clustering and identified a set of genes providing a transcriptomic signature that allows to discriminate between CM and UM children. Even if most of the DEGs were related to expected functions such as erythrocyte alteration, immune/inflammatory response, and brain dysfunction, our analysis provided further insight in disease development and identified novel genes never described in malaria pathogenesis. Secondly, the pathway enrichment analysis revealed that our data were quite robust since some pathways that were dysregulated in previous reports were similarly dysregulated in this study including pathways associated with immune system such as cytokine-cytokine receptor interaction, TNF signaling pathways, and chemokine signaling pathways [10]. Others dysregulated pathways were also consistent with our knowledge of the pathogenesis of CM, as example, the granulocyte adhesion and diapedesis as well as the arginine degradation.

Among the genes that may be involved in the immune response, ARG1 is strongly expressed in CM compared to those with UM which is consistent with previous results. Indeed, previous reports showed that M2-like activation monocyte phenotype was associated with hypoargininemia, NO insufficiency, higher mononuclear cell arginase 1 mRNA, and disease severity in Tanzanian children with *P. falciparum* malaria [20]. It has been proposed that M2 cells produce arginase 1 that converts arginine to ornithine and urea, causing depletion of arginine and reduction of NO. Absence of NO causes increased endothelial cell adhesion molecule expression and adherence of parasitized red blood cell to endothelium, having as a consequence the blockage of blood flow and distal tissue ischemia. In addition, the increased polarization towards the M2 phenotype and the increased arginase activity may also affect the immune response in malaria by altering the metabolism substrates available to lymphocytes and other immune cells. In addition, we showed that new key pathways governing lipid metabolism (LXR/RXR activation) and inflammasome (NOD-like receptor signaling pathway) were altered in our study. Interestingly, NOD-like receptors (NLRs) are a family of cytosolic proteins that play an important role in inflammation and immunity, and besides its established role in inflammation, the LXR/RXR system has emerged as a key regulator of cholesterol, fatty acid and glucose homeostasis, and neuroprotection [21–23]. The pathway and network analysis allow us to confirm that most of DEGs identified here are involved in the immune/inflammatory response and revealed a crucial role of the inflammasome. Inflammasome-mediated secretion of IL-1*β* and IL-18 is aimed at eliminating the infectious pathogen through the induction of secondary mediators and the recruitment of additional immune cells to the infection site and/or pyroptosis of the infected cells. While the inflammasome-mediated response is beneficial for the host, it must be tightly regulated; otherwise, it may be associated with pathology. It is known that IL-18BP is able to bind mature IL-18 and prevents attachment to the IL-18 receptor. Here, we observed higher levels of IL-18 in the plasma of CM children compared to UM and lower expression of IL-18BP in CM suggesting that disease could be associated with an imbalance of IL-18 and IL-18BP resulting in an inappropriate inflammasome activation. Although the level of IL-18 is higher in CM, no significant difference in expression of the transcript was observed between the two groups. It has been described that transcript level and cognate protein level do not necessarily correlate due to the regulation of translation [24]. IL-18/IL-18BP could be considered as a couple with therapeutic potential [25]. This hypothesis is consistent with recent findings showing that IL-33, which can reduce inflammasome activation and IL-1*β* production in microglia, is downregulated in the brain during fatal ECM (experimental CM) [26]. The authors demonstrate that manipulation of the IL-33-NLRP3 axis may be an effective therapy to suppress neuroinflammation and improve the efficacy of antimalarial drug treatment of CM. In addition, specific mutations in inflammasome genes resulting in constitutive or inappropriate activation of the inflammasome have been associated with neurological diseases. Hence, inflammasome has emerging recently as a pathogenic and therapeutic target in neurological diseases [27–29], and we believe that its activation could also have important consequences in CM. Another gene linked to inflammation, AXIN2 is shown to be less expressed in CM and acts as a negative regulator of Wnt/*β*-catenin signaling pathway that plays a critical role in many aspects of cell differentiation and immune cell function [30]. Indeed Wnt/*β*-catenin signaling has been shown to play an important role in the expression of several inflammatory molecules during infections [31], and aberrant signaling has been reported in neurodegenerative disorders such as Alzheimer's and Parkinson's diseases.

Interestingly, the 5 upstream regulators TNF, IL-6, IL-1B, STAT3, and SLC4A1 that we have identified here are all molecules that have already been involved in malaria, and some of them are considered as key players in a diverse mechanism of early innate host defense which are critical for the outcome of *Plasmodium* infection. Our study provided further evidence of their role and allowed the identification of secondary mediators of CM. A total of 55 molecules were shown to be dysregulated by these upstream regulators and then may favor disease development. Although TNF was not differentially expressed here, TNF has been identified by others as a risk factor for the development of severe forms [4, 32–34]. However, its mode of action and causal regulatory variants were not clearly identified because genetic studies showed contradictory results across study populations [7, 35, 36]. Here, it is suggested that TNF could play a key role in pathogenic mechanisms via its regulatory effect on many other molecules. IP-10/CXCL0 is part of these effector molecules. Surprisingly, we found that the IP-10/CXCL10 gene expression and protein levels were higher in UM than in those who recovered from CM, suggesting protective effect, and that these levels were higher in fatal CM compared to CM survivors in favor to a pathogenic role. Consistently with our findings, previous studies showed that IP-10/CXCL10 levels were elevated in fatal CM and were tightly associated with CM mortality [37, 38]. In addition, there is growing evidence that IP-10/CXCL10 plays a role in both infectious and noninfectious causes of CNS neuronal injury, dementia, and inhibition of angiogenesis. IP-10 is an IFN-*γ*-induced chemokine with chemotactic activity for activated Th1 lymphocytes [38] which is quite consistent with the correlation between IFN-*γ* and IP-10 that we observed in our patients. Our results therefore suggested a complex regulation of this gene and a dual role of IP-10 (protective and aggravating) as it has been shown for IFN-*γ* in both malaria [39] and Chagas disease [40]. This regulation can be partly achieved by the upstream regulators identified here, TNF, IL-1B, IL-6, and STAT3. Overall, we can assume that high levels of CXCL10 may cause vascular injury resulting in breakdown in the blood-brain barrier (BBB) which may lead to accumulation of leukocytes that induced local hyperinflammation and to a lethal neuropathological syndrome. On the basis of all these data, we can hypothesize that subjects infected by *P. falciparum* must develop an effective immune response by producing chemokines such as IP-10/CXCL10 and inflammatory cytokines such as IFN-*γ* which can control the infection. Unfortunately, this immune response cannot be properly regulated in some people, and an excessive inflammatory response can cause tissue damages and death. Thus, such excessive production on arrival at the hospital seems to be a diagnosis of poor clinical course. In addition, the kinetics of production of the molecules involved in the inflammatory response and determining in the clinical outcome of the patient as suggested recently for the IFN-*γ* production is essential [41]. However, it remains to be established how this chemokine might contribute to protect against CM.

Finally, this analysis supported again that pathways and genes having a role in the neurodegenerative disorders were dysregulated in CM as we have previously pointed out [8] and which has been confirmed by others [11]. Here, we highlighted the role of 3 genes (GPR88, GPNMB, and GMPR) showing higher expression in CM (FC > 4) and that could be interesting key players of CM since higher expression of them are also associated to Parkinson's and Alzheimer's diseases. These two neurodegenerative disorders share with CM both some clinical symptoms such as memory loss or vasoconstriction and also some pathophysiological features such as a modification of the blood-brain barrier and an increase in the expression of ICAM-1 [42, 43]. The GPR88 is part of the top gene with a fold increase of 9.1. This gene encodes a brain-specific G protein-coupled receptor that plays a role in dopaminergic function. This cell surface receptor which is involved in the recognition of iRBCs (infected red blood cells) [44] is also emerging as a novel drug target for central nervous system disorders including schizophrenia, Parkinson's disease (PD), and anxiety [45]. Another new key player of CM was the glycoprotein nonmetastatic melanoma protein B (GPNMB), a glycoprotein observed upon tissue damage and inflammation and associated with astrocytes, microglia, and macrophages. It has been identified as a novel Alzheimer's disease- (AD-) related factor in both transgenic mice and sporadic AD patients by expression profiling [46]. In addition, gene variations in GPNMB were also linked with PD risk and with significant increased expression of GPNMB [47]. Finally, the GMPR gene, which encodes protein GMPR1, was upregulated in CM as observed in AD cases and which exhibited a gradual increase with AD progression. Importantly, the increased expression of GMPR makes the product of GMPR (GMPR1) a potential therapeutic target [48].

Finally, several studies on gene expression analysis using microarrays showed promising results even by using a small number of samples [10, 16, 49]. Here, despite the limited number of subjects studied for the transcriptome analysis, we obtained sufficient statistical power to identify blood transcriptomic signature which distinguish CM and UM. Obviously, additional studies carried out on a larger number of subjects, and using RNAseq technology would be very useful to confirm the role of some genes identified here.

In conclusion, our study provides fundamental knowledge on the molecular profile characteristic of CM. In this work, we have identified a new set of genes as key signaling and regulatory molecules involved in the pathophysiology of CM. We show that transcriptional profiling in whole blood is powerful enough to allow a comprehensive analysis of host physiology during CM and the identification of biomarkers that may be potential therapeutic targets.

## Figures and Tables

**Table 1:** Significant genes differentiating between CM and UM (∣FC | >2 and adjusted *P* ≤ 0.01).

Probe name	Gene symbol (gene name)	Regulation status	Absolute FC
New key players of cerebral malaria			
A_33_P3380462	GPR88 (G protein-coupled receptor 88)	Up	9.1
A_23_P140675	EPB42 (protein 4.2, erythrocytic)	Up	6.1
A_23_P134426	GPNMB (glycoprotein NMB)	Up	6
A_23_P58266	S100P (S100 calcium-binding protein P)	Up	4.6
A_24_P277657	GMPR (guanosine monophosphate Reductase)	Up	4.1
A_33_P3323559	CRYAA (crystallin, alpha-A)	Up	4
A_23_P321935	OSBP2 (oxysterol-binding protein 2)	Up	4
A_33_P3326225	SAMD12 (sterile alpha motif domain-containing protein 12)	Down	3.6
A_32_P133072	SPON1 (spondin 1)	Down	3.5
A_33_P3402615	SLC6A9^∗^ (solute carrier family 6, member 9)	Up	3.4
A_23_P72077	IL12RB2^∗^ (interleukin 12 receptor beta 2)	Down	3.1
A_23_P392942	MSR1 (macrophage scavenger receptor 1)	Up	2.9
A_23_P102731	SMOX (spermine oxidase)	Up	2.6
A_23_P83931	NET1^∗^ (Neuroepithelial cell transforming gene1)	Down	2.1
A_24_P298027	AXIN2^∗^ (axis inhibitor 2)	Down	2.1
A_33_P3228322	IL18BP^∗^ (interleukin 18 binding protein)	Down	2.1
A_33_P3234530	TNFRSF25 (tumor necrosis factor receptor superfamily, member 25)	Down	2
A_23_P126844			2.2
A_23_P3849	TRAP1 (tumor necrosis factor receptor-associated protein 1)	Down	2

Previously involved in malaria			
A_33_P3343175	CXCL10^∗^ (chemokine, CXC motif, ligand 10)	Down	7.7
A_24_P303091			7.8
A_23_P89431	CCL2 (chemokine, CC motif, ligand 2)	Down	7
A_33_P3416668	VWA1 (Von Willebrand factor A domain-containing protein 1)	Up	6.6
A_21_P0011751	CD177 (CD177 antigen)	Up	5.8
A_24_P63019	IL1R2 (interleukin 1 receptor, type II)	Up	5.2
A_33_P3352382	ARG1^∗^ (arginase 1)	Up	4.6
A_33_P3319967			4.5
A_33_P3376321	ANK1 (ankyrin 1)	Up	3.2
A_23_P216108			3.5
A_23_P127288	IL2RA^∗^ (interleukin 2 receptor, alpha)	Down	3
A_24_P139901	GYPC (glycophorin C)	Up	2.9
A_33_P3214550	CXCR2 (chemokine, CXC motif, receptor 2)	Up	2.6
A_24_P257416	CXCL2 (chemokine, CXC motif, ligand 2)	Up	2.1
A_23_P315364			2.5

Partial list of DEGs included in the RNA signature. Interesting candidate genes identified here as new players of human CM (at the top of the table) or previously involved in malaria (at the bottom of the table). Absolute FC values are shown. The eight genes selected for qPCR validation are indicated by an asterisk (^∗^).

## Data Availability

The data used to support the findings of the study are available from the corresponding author upon request.
